# Multiple drivers of decline in the global status of freshwater crayfish (Decapoda: Astacidea)

**DOI:** 10.1098/rstb.2014.0060

**Published:** 2015-02-19

**Authors:** Nadia I. Richman, Monika Böhm, Susan B. Adams, Fernando Alvarez, Elizabeth A. Bergey, John J. S. Bunn, Quinton Burnham, Jay Cordeiro, Jason Coughran, Keith A. Crandall, Kathryn L. Dawkins, Robert J. DiStefano, Niall E. Doran, Lennart Edsman, Arnold G. Eversole, Leopold Füreder, James M. Furse, Francesca Gherardi, Premek Hamr, David M. Holdich, Pierre Horwitz, Kerrylyn Johnston, Clive M. Jones, Julia P. G. Jones, Robert L. Jones, Thomas G. Jones, Tadashi Kawai, Susan Lawler, Marilu López-Mejía, Rebecca M. Miller, Carlos Pedraza-Lara, Julian D. Reynolds, Alastair M. M. Richardson, Mark B. Schultz, Guenter A. Schuster, Peter J. Sibley, Catherine Souty-Grosset, Christopher A. Taylor, Roger F. Thoma, Jerry Walls, Todd S. Walsh, Ben Collen

**Affiliations:** 1 Institute of Zoology, Zoological Society of London, Regent's Park, London NW1 4RY, UK; 2 School of Environment, Natural Resources and Geography, Bangor University, Bangor, Gwynedd LL57 2UW, UK; 3 USDA Forest Service, Southern Research Station, Center for Bottomland Hardwoods Research, 1000 Front St., Oxford, MS 38655-4915, USA; 4 Colección Nacional de Crustáceos, Instituto de Biología, Universidad Nacional Autónoma de México, Apartado Postal 70-153, México 04510 DF, México; 5 Oklahoma Biological Survey and Department of Biology, University of Oklahoma, Norman, OK 73019, USA; 6 School of Natural Sciences, Edith Cowan University, 270 Joondalup Drive, Joondalup, Western Australia, Australia; 7 Northeast Natural History and Supply, 24 North Grove St., Middleboro, MA 02346, USA; 8 Jagabar Environmental, PO Box 634, Duncraig, Western Australia 6023, Australia; 9 Computational Biology Institute, George Washington University, Ashburn, VA 20147, USA; 10 Department of Invertebrate Zoology, National Museum of Natural History, Smithsonian Institution, Washington, DC 20013, USA; 11 Australian Rivers Institute, Griffith School of Environment, Griffith University, Gold Coast Campus, Queensland 4222, Australia; 12 Missouri Department of Conservation, 3500 East Gans Road, Columbia, MO 65201, USA; 13 Bookend Trust and the School of Biological Sciences, University of Tasmania, PO Box 310, Sandy Bay, Tasmania 7006, Australia; 14 Institute of Freshwater Research, Department of Aquatic Resources, Swedish University of Agricultural Sciences, 178 93 Drottningholm, Sweden; 15 School of Agricultural, Forestry and Environmental Sciences, Clemson University, Clemson, SC 29634, USA; 16 River Ecology and Conservation, Institute of Ecology, University of Innsbruck, Technikerstrasse 25, 6020 Innsbruck, Austria; 17 Griffith School of Environment and the Environmental Futures Research Institute, Griffith University, Gold Coast Campus, Queensland 4222, Australia; 18 Dipartimento di Biologia, Università degli Studi di Firenze, via Romana 17, 50125 Firenze, Italy; 19 Upper Canada College, 200 Lonsdale Road, Toronto, Ontario, Canada M4V 1W6; 20 Crayfish Survey and Research, Peak Ecology Limited, Arden House, Deepdale Business Park, Bakewell, Derbyshire DE45 1GT, UK; 21 Environmental and Conservation Sciences, Murdoch University, 90 South St., Murdoch, Western Australia 6150, Australia; 22 Marine and Freshwater Research Laboratory, Murdoch University, 90 South St., Murdoch, Western Australia 6150, Australia; 23 James Cook University, School of Marine and Tropical Biology, PO Box 6811, Cairns, Queensland 4870, Australia; 24 Mississippi Department of Wildlife, Fisheries, and Parks, Museum of Natural Science, 2148 Riverside Drive, Jackson, MS 39202-1353, USA; 25 Department of Integrated Science and Technology, Marshall University, 1 John Marshall Drive, Huntington, WV 25755, USA; 26 Wakkanai Fisheries Institute, 4-5-15 Suehiro, Wakkanai, 097-0001 Hokkaido, Japan; 27 Department of Environmental Management and Ecology, La Trobe University, Wodonga, Victoria 3690, Australia; 28 Evolutionary Biology and Population Genetics Laboratory, Universidad de Quintana Roo, Unidad Académica Cozumel, Av. Andrés Quintana Roo con Calle 110s/n, Frente a Col. San Gervasio, Cozumel 77600, Q. Roo, México; 29 International Union for Conservation of Nature, Global Ecosystem Management Programme, 219c Huntingdon Road, Cambridge CB3 0DL, UK; 30 Universidad Nacional Autónoma de México, Facultad de Medicina, Circuito Interior, Ciudad Universitaria, Av. Universidad 3000, CP 04510. Universidad Nacional Autónoma de México, Instituto de Biología, tercer circuito s/n, Ciudad Universitaria, Coyoacán, México DF CP 04510, México; 31 Trinity College Dublin, 115 Weirview Drive, Stillorgan, Co. Dublin, Ireland; 32 School of Biology, University of Tasmania, Private Bag 55, Hobart, Tasmania 7001, Australia; 33 Department of Biochemistry and Molecular Biology, University of Melbourne, 30 Flemington Road, Parkville, 3010 Victoria, Australia; 34 305 Boone Way, Richmond, KY 40475, USA; 35 Environment Agency, Wessex Area, Rivers House, East Quay, Bridgwater TA6 4YS, UK; 36 Laboratoire Ecologie et Biologie des Interactions, Université de Poitiers, Equipe Ecologie Evolution Symbiose, UMR CNRS 7267, Poitiers Cedex, France; 37 Prairie Research Institute, Illinois Natural History Survey, 1816 S. Oak, Champaign, IL 61820, USA; 38 Midwest Biodiversity Institute, 4673 Northwest Parkway, Hilliard, OH 43026, USA; 39 Department of Biological Sciences, Louisiana State University Alexandria, 8100 Highway 71 S, Alexandria, LA 71302, USA; 40 34 McKenzie St, Lismore, New South Wales 2480, Australia; 41 Centre for Biodiversity and Environmental Research, University College London, Gower St., London WC1E 6BT, UK

**Keywords:** extinction risk, crayfish, IUCN Red List, threatened, freshwater biodiversity

## Abstract

Rates of biodiversity loss are higher in freshwater ecosystems than in most terrestrial or marine ecosystems, making freshwater conservation a priority. However, prioritization methods are impeded by insufficient knowledge on the distribution and conservation status of freshwater taxa, particularly invertebrates. We evaluated the extinction risk of the world's 590 freshwater crayfish species using the IUCN Categories and Criteria and found 32% of all species are threatened with extinction. The level of extinction risk differed between families, with proportionally more threatened species in the Parastacidae and Astacidae than in the Cambaridae. Four described species were Extinct and 21% were assessed as Data Deficient. There was geographical variation in the dominant threats affecting the main centres of crayfish diversity. The majority of threatened US and Mexican species face threats associated with urban development, pollution, damming and water management. Conversely, the majority of Australian threatened species are affected by climate change, harvesting, agriculture and invasive species. Only a small proportion of crayfish are found within the boundaries of protected areas, suggesting that alternative means of long-term protection will be required. Our study highlights many of the significant challenges yet to come for freshwater biodiversity unless conservation planning shifts from a reactive to proactive approach.

## 1. Introduction

Freshwater ecosystems occupy less than 1% of the earth's surface, but support approximately 10% of the world's species and 30% of all vertebrates [1]. These systems provide a range of valuable services, including fisheries, domestic and commercial water supply, carbon sequestration and energy; however, a rapidly growing human population has increased the demand on freshwater resources leading to a freshwater biodiversity crisis [2]. While knowledge on the conservation status and distribution of freshwater taxa is disparate relative to terrestrial species [3], there is growing evidence that freshwater taxa (i.e. crabs, dragonflies, fish and molluscs) are at greater risk of extinction than terrestrial vertebrates (i.e. mammals, reptiles or birds) [3–9]. Given the disproportionately high biodiversity harboured in freshwater ecosystems, knowledge on the distribution and conservation status of freshwater species will be essential for monitoring targets set by the Convention on Biological Diversity [3]. For example, Target 6 aims to ensure that ‘all fish and invertebrate stocks and aquatic plants are managed and harvested sustainably by 2020’, Target 11 is to conserve 17% of inland water by 2020 and Target 12 requires that by 2020 ‘the extinction of known threatened species has been prevented and their conservation status, particularly of those most in decline, has been improved and sustained’ [10].

Limited resources available for conservation require practitioners to prioritize areas for action. Selection of priority areas requires knowledge on the distribution and conservation status of a globally representative sample of species. To date, global analyses of species diversity and patterns of threat have been biased towards terrestrial species, particularly vertebrates [11–13] producing the major tropical and subtropical hotspots described by Myers *et al*. [11]. However, there is growing evidence that vertebrates are a poor proxy for estimating invertebrate diversity [3,14,15], highlighting a need for improved knowledge on the distribution and status of invertebrate taxa.

Freshwater crayfish (Astacidea) exhibit a disjunct global distribution with the majority of species diversity restricted to temperate latitudes, and an absence of native species in continental Africa and the Indian subcontinent [16]. A number of hypotheses explaining crayfish distribution patterns have been proposed: competitive exclusion with the freshwater crabs that occupy a similar ecological niche [17–19]; unsuitable climatic conditions [17,19,20]; or the timing of the separation of Gondwana [16]. However, these hypotheses have been neither denied nor supported, and so an explanation for the absence of crayfish in Africa and India remains inconclusive.

The major crayfish diversity hotspots are split taxonomically into two superfamilies: Astacoidea and Parastacoidea [21]. Astacoidea is restricted to the Northern Hemisphere and comprises two families: Cambaridae, which is the largest crayfish family and native to North America (409 spp.) and East Asia (four spp.); and Astacidae, the smallest family, with native species in Europe (five spp.) and the USA and Canada (five spp.). Parastacoidea comprises only a single family, the Parastacidae, which is restricted to the Southern Hemisphere [15] with native species in Australasia (148 spp.), Madagascar (seven spp.) and South America (12 spp.).

Crayfish are found in a diversity of habitats, including: permanent and seasonal rivers, streams and lakes; freshwater caves and springs; and terrestrial burrows. Given their significant biomass in many freshwater systems [22], crayfish play a fundamental role in determining ecosystem structure and function [23], and are of significant economic importance, particularly in Madagascar, Europe, China and the US state of Louisiana [24–26]. However, in recent years, freshwater crayfish have been increasingly recognized as in need of ‘conservation attention’ [27,28]. Previous estimates suggest that 48% of North American crayfish species and 25% of all Australian species are threatened [27–29], and that extinction rates for crayfish may increase by more than an order of magnitude exceeding those of freshwater fishes and amphibians [8]. Heightened extinction risk in crayfish is often attributed to small range size and degradation of freshwater habitats [30]; however, even the wide-ranging European noble crayfish (*Astacus astacus*) has seen significant population declines since the arrival of crayfish plague (*Aphanomyces astaci*) [31].

Threats to crayfish are set to increase in both magnitude and extent. Consequently, there is an urgent need to better understand the extinction risk and patterns of threat in freshwater crayfish. In this study, we address these gaps by assessing the global extinction risk of all crayfish species described up to 2009, using the International Union for Conservation of Nature (IUCN) Red List of Threatened Species Categories and Criteria [32]. We report on patterns of extinction risk across families, analyse patterns of threat and data gaps, and make recommendations for conservation.

## 2. Methods

Species-specific data were collected on taxonomy, distribution, population trends, ecology, biology, threats and conservation measures for all 590 species of crayfish described up to 2009. Data were obtained from published and unpublished articles, government reports and personal communications. All species were evaluated against quantitative thresholds defined in the IUCN Red List Categories and Criteria [33] to assess extinction risk based on: A (past, present or future declining population), B (geographical range size, and fragmentation, decline or fluctuations), C (small population size and fragmentation, decline or fluctuations), D (very small population or very restricted distribution) and E (quantitative analysis of extinction risk). Based on the quantitative thresholds and available data, we assigned one of the eight IUCN Red List categories [32]: Extinct (EX), Extinct in the wild (EW), Critically Endangered (CR), Endangered (EN), Vulnerable (VU), Near Threatened (NT), Least Concern (LC) and Data Deficient (DD), of which CR, EN and VU are the threatened categories. Few invertebrate species have sufficient information on rates of population decline, so assessments under criterion A were based on presence/absence data over time, assuming equal abundance across the range and linear rates of decline. Following Darwall *et al*. [34], we mapped species distributions to river sub-basins as delineated by the HYDRO1k Elevation Derivative Database [35] using ArcGIS v. 9.3. Where existing distribution maps were available these were digitized, while others were created from georeferenced specimen collection records provided by species experts. We calculated species range either as: extent of occurrence (EOO), by computing a minimum convex polygon around all known, inferred and projected occurrences; or area of occupancy (AOO), by calculating the area of all known occupied sites. Species assessments and distribution maps were reviewed by a panel of experts in a workshop setting, and remotely by email. The majority of assessments (*n* = 573) were published on the IUCN Red List in 2010, with 17 assessments awaiting publication.

Following Hoffmann *et al*. [36], we estimated the proportion of threatened species as [(number of threatened)/(total − DD)], where ‘threatened’ is the number of species assessed VU, EN and CR, ‘total’ is the total number of species and DD is the number of species assessed as DD. This assumes that DD species show the same proportion of threatened species as better known species, and represents a mid-estimate of extinction risk for the group (see [31]). Threat levels have been reported this way in similar studies [6,13,36], representing the current consensus among conservation biologists about how the proportion of threatened species should be presented, while also accounting for the uncertainty introduced by DD species. We also calculated a lower estimate on the proportion of threatened species assuming that none of the DD species are threatened [(number of threatened)/total] and a high estimate assuming that all DD species are threatened [(number of threatened + DD)/total]. Extinction risk was summarized across all families and genera.

Identification of taxa that are more threatened than expected by chance can help prioritize conservation actions [37]. Using the methods described by Bielby *et al*. [38], we tested to see whether genera deviated from the expected level of threat. Chi-squared tests were used to test for significant departures from equal threat between genera, and binomial tests were used to find the smallest genus size necessary to detect a significant deviation from the observed proportion of threatened species. Genera represented by an insufficient number of species were excluded. A null frequency distribution of the number of threatened species was generated from 10 000 unconstrained randomizations, by randomly assigning Red List categories to all species, based on the frequency of occurrence of each category in the sample. The number of threatened species in the focal genera was counted and compared with the null frequency distribution. The null hypothesis (that extinction risk is taxonomically random) was rejected if this number fell in the 2.5% at either tail of the null frequency distribution.

Following Salafsky *et al*. [39], threats were categorized into: agriculture, logging, invasive species and disease, problematic native species, harvesting, urban development (i.e. commercial, domestic and industrial), energy production and mining, climate change and severe weather events, pollution, human disturbance (i.e. war and recreational activities), transportation infrastructure (i.e. roads, shipping lanes, railways) and water management/dams. Threats were summarized by geographical location only for threatened species.

We assessed the spatial congruence between threatened species richness and DD species richness in the major centres of diversity (i.e. Australia, Mexico and the USA). We defined centres of richness by selecting the top 10% species-rich river basins, with richness based on the absolute number of species, DD species and threatened species and compared congruence using Pearson's correlations. We accounted for spatial autocorrelation by implementing the method of Clifford *et al*. [40], which estimates effective degrees of freedom based on spatial autocorrelation in the data and applies a correction to the significance of the observed correlation. We also assessed the proportions of southeast US and Australian threatened species’ basins that intersect with protected areas (irrespective of the proportion of the basin area covered). Protected areas were selected using the IUCN Protected Areas Categories System [41], and included the following categories: strict nature reserve, wilderness area, national park, natural feature, habitat/species management area, protected landscape and protected area with sustainable use of natural resources. All statistical analyses were performed using the software package R v. 3.0.1 [42]. The critical value for *α* was set at 0.05.

## 3. Results

Nearly one-third of the world's crayfish species were assessed as threatened with extinction assuming that DD species are threatened in an equal proportion (32%: range 24–47%; table 1). Of the non-threatened species, 7% were assessed as NT and 47% as LC. Twenty-one per cent of all species were assessed as DD. Four species were assessed as EX; however of the 51 species assessed as CR, four were highlighted as possibly extinct. Of the EX species, two were previously found in Mexico (*Cambarellus alvarezi* and *Cambarellus chihuahuae*) and two in the USA, specifically Georgia (*Procambarus angustatus*) and California (*Pacifastacus nigrescens*). Of the possibly extinct species, two were known from Mexico (*Procambarus paradoxus* and *Cambarellus areolatus*), and one each from the US states of Alabama (*Cambarus veitchorum*) and Florida (*Procambarus delicatus*). All East Asian *Cambaroides* and South American Parastacidae (10 of 12 spp.) were assessed as DD. Only two of the seven species of Malagasy *Astacoides* were assessed as threatened, whereas the remaining species were assessed as DD (four of seven spp.) or LC (one of seven spp.).

**Table 1.** Extinction risk summarized by family and genus. Figures for the proportion of threatened species represent the mid-estimate [(number of threatened)/(total−DD)], lower estimate [(number of threatened)/total] and high estimate [(number of threatened + DD)/total].

taxa	native geographical locality	DD	LC	NT	VU	EN	CR	EX	total	proportion threatened (low estimate–high estimate)
**Astacidae**		**3**	**3**	**0**	**1**	**1**	**1**	**1**	**10**	**43% (30–60%)**
*Astacus*	Europe	1	1	0	1	0	0	0	3	50% (33–67%)
*Austropotamobius*	Europe	1	0	0	0	1	0	0	2	100% (50–100%)
*Pacifastacus*	USA, Canada	1	2	0	0	0	1	1	5	25% (20–40%)
**Cambaridae**		**91**	**221**	**26**	**20**	**33**	**19**	**3**	**413**	**22% (17–39%)**
*Barbicambarus*	USA	0	1	0	0	0	0	0	1	0% (0–0%)
*Bouchardina*	USA	1	0	0	0	0	0	0	1	0 (0–100%)
*Cambarellus*	USA, Mexico	3	8	1	0	1	2	2	17	21% (18–35%)
*Cambaroides*	East Asia	4	0	0	0	0	0	0	4	0% (0–100%)
*Cambarus*	USA, Canada	15	61	9	4	5	7	0	101	19% (16–31%)
*Distocambarus*	USA	3	0	0	2	0	0	0	5	100% (40–100%)
*Fallicambarus*	USA, Canada	2	8	5	1	1	1	0	18	19% (17–28%)
*Faxonella*	USA	0	3	1	0	0	0	0	4	0% (0–0%)
*Hobbseus*	USA	3	1	0	0	3	0	0	7	75% (43–86%)
*Orconectes*	USA, Canada, Mexico	9	62	3	10	4	1	0	89	19% (17–27%)
*Procambarus*	USA, Mexico, Cuba, Belize, Guatemala, Honduras	51	77	6	3	19	8	1	165	26% (18–49%)
*Troglocambarus*	USA	0	0	1	0	0	0	0	1	0% (0–0%)
**Parastacidae**		**31**	**50**	**14**	**12**	**33**	**27**	**0**	**167**	**53% (43–62%)**
*Astacoides*	Madagascar	4	1	0	0	2	0	0	7	67% (29–86%)
*Astacopsis*	Australia	0	2	0	0	1	0	0	3	33% (33–33%)
*Cherax*	Australia, New Guinea	9	12	6	2	7	3	0	39	40% (31%–54%)
*Engaeus*	Australia	5	17	3	3	3	4	0	35	33% (29–43%)
*Engaewa*	Australia	0	2	0	0	2	1	0	5	60% (60–60%)
*Euastacus*	Australia	1	8	1	5	17	17	0	49	81% (80–82%)
*Geocharax*	Australia	0	1	0	1	0	0	0	2	50% (50–50%)
*Gramastacus*	Australia	0	0	1	0	0	0	0	1	0% (0–0%)
*Ombrastacoides*	Australia	2	4	2	1	0	2	0	11	33% (27–45%)
*Paranephrops*	New Zealand	0	2	0	0	0	0	0	2	0% (0–0%)
*Parastacus*	South America	6	1	1	0	0	0	0	8	0% (0–75%)
*Samastacus*	South America	1	0	0	0	0	0	0	1	0% (0–100%)
*Tenuibranchiurus*	Australia	0	0	0	0	1	0	0	1	100% (100–100%)
*Virilastacus*	South America	3	0	0	0	0	0	0	3	0% (0–100%)
**all species**		**125**	**274**	**40**	**33**	**67**	**47**	**4**	**590**	**32%** (**24–47%**)

The majority (117 of 147 spp.) of threatened species (those classified as CR, EN or VU) were assessed using criterion B1 (geographical range size combined with fluctuations or declines). Only 13 species had adequate surveys from which to calculate AOO and thereby carry out assessments under criterion B2. Five species were assessed under criterion A (*Astacus astacus, Austropotamobius pallipes, Astacopsis gouldi*, *Cambarus cracens* and *Engaeus granulatus*); the other species had insufficient data on rates of population decline to meet this criterion. The assessment for *Astacus astacus* was based on population data from both systematic surveys and direct exploitation, whereas the other assessments were based on observed declines in EOO and AOO collected from systematic surveys over significant parts of the species' ranges. The remaining 12 threatened species were assessed under criterion D2 (i.e. species with a very small range—AOO <20 km^2^ or <5 locations—and subjected to rapidly becoming CR or EX as a result of future threat(s)). A minimum of three species in a genus were required to establish if the genera was at greater risk of extinction than expected by chance, and 10 species per genera to establish if the genera was less threatened than would be expected. This resulted in the exclusion of 18 of 30 genera from the analysis. Extinction risk was non-randomly distributed among genera (*χ*^2^ = 61.15, *p* < 0.001, d.f. = 28) with 11 of the remaining genera being more threatened than expected (table 2). Only the genus *Cambarus* showed a non-significant difference between the proportions of expected and observed threatened species.

**Table 2.** Threat distribution across genera for which there were sufficient samples to determine whether species were more threatened than would be expected by chance, or under threatened: n.s., not significant; +, over threatened; −, under threatened.

family	proportion observed	proportion expected	total species (non-DD)	>expected threat level *p*-value	<expected threat level *p*-value	over or under threatened
*Pacifastacus*	0.333	0.009	3	<0.001	1	+
*Cambarellus*	0.250	0.028	12	<0.001	1	+
*Cambarus*	0.186	0.171	86	0.282	0.718	n.s.
*Fallicambarus*	0.188	0.031	16	<0.001	1	+
*Hobbseus*	0.750	0.012	4	<0.001	1	+
*Astacoides*	0.667	0.012	3	<0.001	1	+
*Astacopsis*	0.333	0.005	3	<0.001	1	+
*Cherax*	0.400	0.066	30	<0.001	1	+
*Engaeus*	0.333	0.059	30	<0.001	1	+
*Engaewa*	0.600	0.009	5	<0.001	1	+
*Euastacus*	0.813	0.083	48	<0.001	1	+
*Ombrastacoides*	0.333	0.019	9	<0.001	1	+

**Figure 1.**
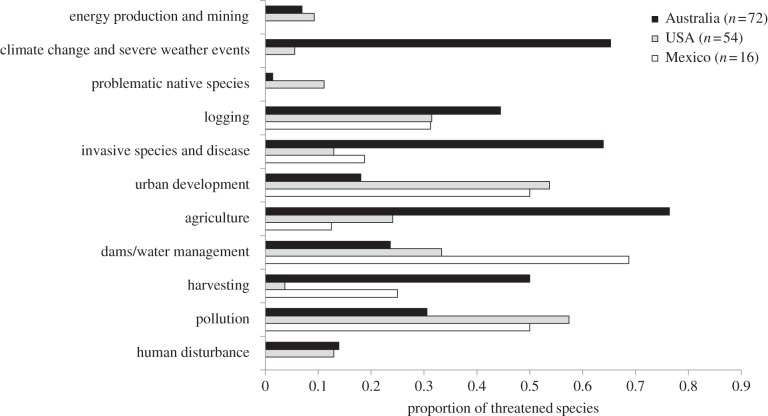
Global threats affecting threatened species within the species-rich (>10 species) geographical regions.

Sixty-five per cent of Australian threatened species were predicted to be at risk from climate-related threats, compared with only 5% of North American species. Similarly, invasive species, disease, agriculture and harvesting were found to impact a greater proportion of Australian threatened species than for Mexican and USA species. Threatened USA species were at greater threat from factors resulting in degradation and loss of habitat, notably urban development and pollution (figure 1). A similar pattern was observed in threatened Mexican species, but with dams and water management impacting a greater proportion of species. For Malagasy species, dominant threats were similar to those described for Australian species: invasive species, agriculture (i.e. land conversion for rice paddies) and harvesting but with no threat from climate change. On average, USA species were found to face fewer threats per threatened individual crayfish (2.1) than Mexican (2.2), Australian (3.9), Malagasy (4) and European (8) threatened species.

**Figure 2.**
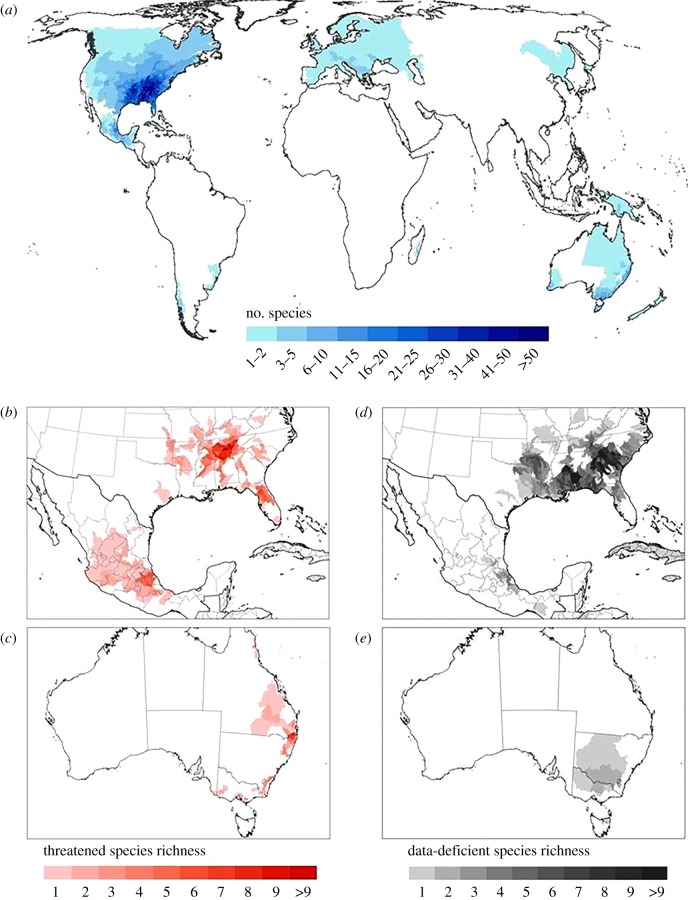
Distribution of: (*a*) all species; (*b*) North American threatened species; (*c*) Australian threatened species; (*d*) North American data-deficient species; and (*e*) Australian data-deficient species. (Online version in colour.)

Crayfish were recorded in 60 countries, with 98% of species found to be endemic to a single country (562 of 590 spp.). In the USA, the major hotspot of diversity is in the southeast USA (notably Tennessee, Alabama and Mississippi; figure 2*a*) where 53% of species (189 of 357 spp.) are known from a single state. In Mexico, 95% (3 of 54 spp.) of species are endemic to the country with a major hotspot of diversity in the Gulf of Mexico region (figure 2*a*). In Australia, 84% (109 of 130 spp.) of species were found in only a single state with hotspots of diversity in the southeast and eastern Australia (southeast Victoria, Tasmania, northeastern New South Wales and southeastern Queensland; figure 2*a*). Distribution of threatened species richness (figure 2*b,c*) largely mirrors total species richness with higher numbers of threatened species in Australia (*n* = 60) than the USA (*n* = 56) or Mexico (*n* = 16). Numbers of DD species were highest in the USA (particularly Tennessee, South and North Carolina, the Florida Panhandle and Mobile River basin) and the Gulf of Mexico region (figure 2*d*) with 85% of DD species having an EOO of less than 20 000 km^2^. We observed relatively few DD species in Australia (figure 2*e*).

There was no correlation between data deficiency and centres of threatened species richness in Australia (*r* = 0.11, *p* = 0.60, d.f. = 24) or Mexico (*r* = 0.60, *p* = 0.086, d.f. = 710). However, there was a marginally non-significant correlation between data-deficiency and threatened species richness in the USA (*r* = 0.21, *p* = 0.06, d.f. = 141). There was low spatial overlap for both the USA (2%) and Australian (6.6%) threatened species and protected areas.

## 4. Discussion

### (a) Patterns of threat and extinction risk

We found nearly one-third of the world's crayfish species are threatened with extinction. This level of threat exceeds that of most terrestrial and marine taxa, but is similar to that of the freshwater crabs and amphibians [5–7,13,43–45], highlighting the imperilled status of freshwater species. The taxonomically non-random distribution of extinction risk in crayfish suggests that certain intrinsic biological traits and external geographical factors might combine to influence risk. However, understanding the factors that drive high extinction risk and the synergistic effect of threats is complicated by a lack of spatial overlap between families [46], and by geographical variation in dominant threats; the biological traits that predict high risk under one threat type may not do so under another threat [47]. Notable differences in extinction risk between the genera of the Australian Parastacidae and the North American Cambaridae might be explained by levels of trait diversity, with both exhibiting considerable trait diversity across genera. For example, Parastacidae genera known only from Australia tend to exhibit small highly fragmented ranges, whereas South American and New Zealand genera exhibit large contiguous ranges (more than 20 000 km^2^). Differences in range size might be explained by the cooler climatic conditions of the Late Cretaceous and widescale flooding in both South America and New Zealand [48–50] both of which have facilitated crayfish dispersal. However, the Australian species-rich genera exhibit low trait diversity within genera, relative to genera of the Cambaridae [51]. For example, slow growth, apparent limited tolerances to increased temperatures [52], late sexual maturity and/or restricted ranges are all characteristic traits of the Australian genus *Euastacus* [53] (traits that tend to predict high risk of extinction in other taxa [33,34,54]), whereas the Australian *Gramastacus* and *Geocharax* are relatively small, have short lifespans and early sexual maturity, and can tolerate a wide range of environmental conditions as they occur in permanent and ephemeral freshwater systems [55]. Conversely, species of the North American genus *Orconectes* range from the cave-dwelling and long-lived (approx. 22 years) southern cave crayfish (*Orconectes australis* [56]), to the river- and lake-dwelling invasive spiny-cheek crayfish (*Orconectes limosus*) which lives for only 4 years [57].

Differences in the level of extinction risk between crayfish families might be partly explained by taxon age. A recent study of the world's marine lobsters dated the origin of Parastacidae to approximately 260 Ma and Cambaridae to approximately 160 Ma [58]. Older taxa might be expected to exhibit higher levels of extinction risk as all taxa must eventually go extinct [59]. A positive relationship between taxon age and extinction risk has been observed in birds [60]. However, in South Africa, the opposite relationship has been observed in plants where extinction risk is greater in the younger taxa [61]. The authors attribute this to the inherently small range size of rapidly diversifying lineages, a key trait for assessing extinction risk using the IUCN Red List Categories and Criteria [33]. There has been rapid diversification in the Cambaridae, resulting in 12 genera and 413 species (at the time of assessment; species lists are still growing), relative to the older Parastacidae (14 genera and 167 species). Congruence between areas of high human density and crayfish diversity might explain why the only known recent crayfish extinctions are from the USA and Mexico. With human density projected to increase within North America [62], continued loss and degradation of habitat (namely urban development, pollution, damming and water management) is likely not only to increase extinction rates but to impede future diversification.

While human density is lower in Australia than North America [62], Australian species face on average a greater number of threats. This complicates identifying the contribution of each threat to rates of decline as many threats act synergistically. For example, increasing temperatures and land conversion from natural state to agricultural use have increased the rate of irrigation, prompting water shortages and salinization of freshwater wetlands [63]. Similarly, increased logging of mature forests has increased the frequency of forest fires in southeast Australia [64]. While threats acting independently of one another may pose little danger to a species, threats acting synergistically can significantly increase rates of decline. In a recent study [65], declines in the population size of rotifers were 50 times faster when threats acted together. Uncertainty in the nature of dependency between threats poses a significant challenge to the effective allocation of conservation resources, and therefore may require action on multiple threats simultaneously.

Of all the geographical localities, European crayfish face the greatest number of threats, of which the most widespread is invasive species. Despite their large geographical ranges, declines of between 50% and 80% have been observed in the white-clawed crayfish (*Austropotamobius pallipes*) [66], and 50% and 70% in the noble crayfish (*Astacus Astacus*) [67]. The effect of interacting threats is particularly evident in the northern part of both species' ranges where populations have disappeared as rising temperatures have facilitated the range expansion of signal crayfish (*Pacifastacus leniusculus*) [68] and crayfish plague (*Aphanomyces astaci*) [69]. At present, invasive crayfish are not a widespread threat across the USA, although the invasive rusty crayfish (*Orconectes rusticus*) is currently expanding its range by up to 30 km per year [70]. The threat of invasive species was most evident in Australia, though invasive crayfish are a relatively minor threat relative to other species. Most of the *Euastacus* species are threatened by invasive predators such as cane toads (*Rhinella marina*) and feral pigs (*Sus scrofa*) which prey on young crayfish and destroy riparian habitat [53]. While invasive species are a prevalent threat to Australian crayfish, the impact of invasive species was often only attributed to localized declines [53].

### (b) Deficits in knowledge

A high proportion of DD species can create taxonomic and geographical biases in the knowledge of extinction risk and the distribution of threat [46]. The proportion of DD crayfish was relatively similar to many previously assessed vertebrate groups (mammals, reptiles, amphibians and fish) [36], but low compared with other invertebrates, such as the freshwater crabs, dragonflies and freshwater molluscs [5–7]. Improved knowledge on the status of DD species is unlikely to significantly alter spatial patterns of extinction risk in the crayfish as there is already high spatial overlap between threatened and DD species in North America, and there are only small numbers of DD species elsewhere. However, the spatial overlap between threatened and DD North American species means there could be many more threatened species. An advantage of this close proximity means opportunities may exist to collect data on DD species while carrying out surveys on better known species, or species receiving survey attention because of conservation concern. Similarly, actions taken to protect better known species may positively benefit a number of these DD species. The majority of North American DD species have ranges smaller than 20 000 km^2^ and so may qualify for a threatened assessment under criterion B, if they are also found to be undergoing declines or fluctuations. However, a lack of information on whether threats are driving declines or fluctuations in range size, number of mature individuals or habitat quality prevented a threat assessment. There are entire genera for which there is little information on population trends, namely the *Samastacus*, *Virilastacus* and *Cambaroides*. Many of these species exhibit large continuous ranges and are therefore unlikely to qualify for a threat assessment under criteria B or D: threat assessments would only be possible under criterion A which would require detailed information on rates of population decline, or data sources from which to derive adequate proxies.

### (c) Conservation

Despite the growing evidence for a freshwater biodiversity crisis, freshwater species remain a low priority on the conservation agenda. Freshwater species, particularly invertebrates, continue to be under-represented within protected area networks. In Africa, approximately one-third of threatened freshwater molluscs and freshwater crabs have 70% or more of their catchments within a protected area, compared with 75% of birds and 98% of mammals [3]. In this study, we observed even fewer crayfish within the boundaries of protected areas. Furthermore, our analysis was based on species ranges intersecting with protected areas which will overestimate the value of protected areas [71], so the proportion of species with greater than 70% of their catchments within protected area boundaries is almost certainly less. Even where species are within protected areas, these are unlikely to be managed for the preservation of freshwater biodiversity [72].

Similarly, freshwater invertebrates are under-represented on national endangered species lists. In the USA, 20% of mammals are listed on the Endangered Species Act list, compared with only 9% of molluscs and 1% of crayfish [73]. In Australia, 25% of terrestrial mammals are listed on the Environment Protection and Biodiversity Protection Act list, but only 5% of freshwater bivalves and 9% of crayfish [74]. Establishing effective conservation actions for many of the more threatened species is made complicated by the types of habitats occupied by some species. Many of the more threatened crayfish and freshwater molluscs are found in intermittent water bodies. Intermittent streams can support distinct and diverse biological communities, but despite their prevalence in the USA [75] they receive no protection under the US Clean Water Act [76].

Conservation of freshwater biodiversity is partly impeded by an inadequate understanding of the economic value of freshwater species and the services they provide [7]. To date, the majority of conservation effort is targeted towards charismatic species or those with a recognized economic value [77]. However, an economic valuation of biomes found freshwater systems were 34 times more valuable than terrestrial systems per unit area [78]. While placing an economic value on nature has its risks [79], realistic economic valuations of freshwater biodiversity and its services could be an important tool for moving freshwater conservation up the agenda.

Incorporating economics into conservation planning will aid the development of cost-effective measures. Conservation costs increase with extinction risk [80], and so actions focused on prevention rather than mitigation could present significant cost-saving opportunities. Invasive species are predicted to significantly increase extinction rates over the next century [81]. Every year, invasive species cost the USA economy $138 billion [82]. While the cost of eradication and control is often significantly higher than the cost of prevention [83], invasive species prevention is greatly under-funded [84]. A recent study estimated the cost of preventing zebra mussel (*Dreissena polymorpha*) invasion into one USA lake at $324 000 a year [84]. At present, the US Fish and Wildlife Service allocates $825 000 for the control and prevention of all invasive species in all lakes across the USA [84]. While it is not feasible to prevent invasion at all sites, not all sites are vulnerable to invasion. Prioritizing sites for protection from invasive species requires knowledge on the mechanisms of species colonization, suitability of habitat for invasive species, and the potential impact of the species [85]. A recent study employed machine learning methods for predicting sites most vulnerable to biological invasion by crayfish [85]. Methods such as these could be used to prioritize sites for protection by identifying hotspots of freshwater diversity that are most vulnerable to invasion by a range of aquatic invaders.

It is unlikely that actions against climate change can be implemented in a timescale that would avert significant biodiversity loss. A key strategy for tackling the effect of climate change will require the maintenance of ecological resilience—that is, the capacity of an ecosystem to withstand or recover from disturbance [86]. For many freshwater species, this will require maintenance of natural connectivity between freshwater habitats allowing for distributional shifts in response to changing environmental conditions. Two-thirds of Australian crayfish species are at risk from climate-mediated threats, a threat that is exacerbated by poor connectivity between areas of suitable habitat. However, identifying species most at risk is impeded by a lack of data on species' thermal limits and environmental parameters (e.g. moisture availability and temperature) [87]. Studies are needed to establish thermal tolerances in crayfish, whether thermal stress is already evident in Australian species, and establish current environmental parameters (primarily temperatures) for a representative selection of Australian ‘indicator’ species. These indicator species should include ‘at risk’ species from the various genera, and include the CR species of *Euastacus* that have been previously identified as ancient ‘climate refugees’ [52]. It would be prudent to develop management plans for the most CR species, and the need to consider maintaining captive populations and/or the relocation of species to more suitable habitats might be unavoidable given the nature and scale of the threats. With climate change now identified as one of the most significant threats affecting Australian freshwaters, developing baseline levels for a range of freshwater environmental parameters has been identified as a research priority [6,87–90]. Without action, it is predicted that climate change will increase in extent and intensity over the next century [91], and so many of the research gaps discussed here need to be considered in other freshwater biodiversity hotspots. Without efforts to address these data gaps, identification of ‘at risk’ species will be difficult and will limit future efforts to protect the ecological integrity of freshwaters.

This study highlights the major research gaps that hamper effective conservation planning for crayfish, many of which would positively benefit a range of freshwater taxa. Conservation planning needs to shift from a reactive to proactive approach if we are to safeguard freshwater systems against anthropogenic environmental damage.
